# The Usefulness of Magnetic Resonance Imaging (MRI) for the Detection of Local Recurrence after Mastectomy with Reconstructive Surgery in Breast Cancer Patients

**DOI:** 10.3390/diagnostics12092203

**Published:** 2022-09-11

**Authors:** Jeongmin Lee, Bong Joo Kang, Ga Eun Park, Sung Hun Kim

**Affiliations:** Department of Radiology, College of Medicine, Seoul Saint Mary’s Hospital, The Catholic University of Korea, Seoul 06591, Korea

**Keywords:** breast cancer, mastectomy, breast reconstruction, local recurrence, breast MRI

## Abstract

The purpose of this study is to investigate the usefulness of magnetic resonance imaging (MRI) for the detection of local recurrence after nipple-sparing mastectomy (NSM) or skin-sparing mastectomy (SSM) with immediate reconstructive surgery for breast cancer. Two hundred and eighty-six NSM or SSM procedures and immediate reconstruction cases between August 2015 and February 2020 were reviewed. The detectability rates of for local recurrence using MRI and ultrasound were assessed, and the characteristics of recurrent and primary cancers were evaluated. The patients with multifocal or multicentric primary cancer and a dense parenchymal pattern showed a higher recurrence rate (*p* < 0.001). A total of 22 cases showed recurrence, and due to multifocal recurrence, a total of 27 recurrent lesions were identified in the reconstructed breast, of which 12 were symptomatic and 15 were asymptomatic (*p* < 0.001). With the exception of skin recurrence (*n* = 6), MRI showed a significantly higher detectability rate (95.2%, 20 of 21) than ultrasound (38.1%, 8 of 21) for the recurrence of cancer in the reconstructed breast (*p* < 0.001), especially for small-sized (<1 cm) asymptomatic lesions. In addition, the mean recurrence interval of MRI-detected asymptomatic lesions was 21.7 months (SD ± 17.7), which was significantly longer than that of symptomatic recurrence. In conclusion, postoperative MRI can be useful for identifying small-sized (<1 cm) asymptomatic recurrence lesions in reconstructed breast tissue after NSM or SSM, which can be implemented within two years of surgery.

## 1. Introduction

Breast cancer is the most common malignancy among Korean women, with a rapidly increasing incidence rate [1]. With this trend in mind, surgical techniques of breast cancer surgery have been developed over recent decades, and the preferred surgical methods have changed constantly. The rate of mastectomy has increased, and the rate of reconstructive surgery after mastectomy has been increasing in Korea [2] as well as the United States [3,4]. In Korea, the number of reconstructive surgeries after mastectomy has continued to rapidly increase, as the National Health Insurance System has been covering the costs of reconstructive surgery after total mastectomy since 2015 [5]. 

Recently, the rate of nipple-sparing mastectomy (NSM) or skin-sparing mastectomy (SSM), as methods for optimal reconstructive surgery after mastectomy, has increased [6,7]. Both techniques excise the entire breast tissue while preserving the skin envelope and natural inframammary fold. In addition, NSM preserves the nipple–areolar complex [8,9,10]. The cosmetic outcome of these surgeries may be better than that of the conventional mastectomy. However, the main concern regarding these surgical techniques is their oncologic safety in relation to the possibility of the remaining breast tissue including tumor cells, which could result in local recurrence [11,12,13]. Moreover, immediate reconstruction followed by NSM or SSM is related to concerns regarding the delayed diagnosis of local or loco-regional recurrence [14].

Despite the increasing number of immediate reconstructive surgeries after mastectomy, there are no available guidelines for postoperative surveillance after mastectomy [15,16,17]. Clinical surveillance, including physical examination, has been widely accepted as a method of caring for patients who undergo total mastectomy without reconstruction, and some previous studies demonstrated the reliability of physical examination in the care of those patients [18,19,20]. However, the physical examination of reconstructed breasts is less sensitive than that that of non-reconstructed breasts due to variable postoperative changes resulting from reconstruction [21,22]. 

Mammography has been recommended as the only surveillance modality for use after breast cancer treatment, because mammography enables the detection of an asymptomatic recurrence and enables early treatment [23,24]. However, the concept of applying mammography to reconstructed breasts is controversial. From previous studies [25,26], we find that rates of recurrences detected by mammography are comparable to those detected by physical examinations in one previous study. In addition, the mammography of reconstructed breasts is not advantageous for the evaluation of disease recurrence, because it is suboptimal due to the post-surgical distortion of reconstructed breasts and poor compliance for mammography positioning.

Breast MRI, as the most sensitive imaging modality for the detection of breast malignancies, has been shown to be useful as a surveillance method after breast-conserving surgery in previous studies [27,28,29]. However, there are few data regarding the usefulness of breast MRI after reconstructive surgery and mastectomy. Therefore, we planned to investigate the usefulness of postoperative MRI for detecting local recurrence in reconstructed breasts. In addition, characteristics of recurrent lesions, according to the presence of symptoms, were also investigated. 

## 2. Materials and Methods 

### 2.1. Subjective 

This retrospective study was approved by the institutional review board (IRB) of our institution, and informed consent was waived by the ethics committee due to its retrospective design. All procedures involving human participants were in accordance with the ethical standards of IRB of our institution, and assessments were carried out as per the rules of the Declaration of Helsinki of 1975, revised in 2013. 

**Figure 1:**
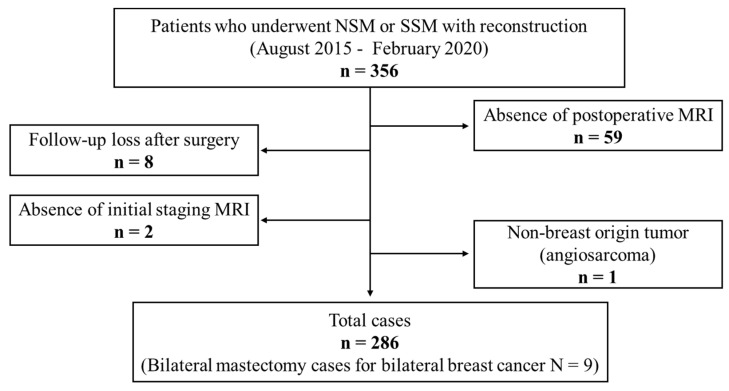
Flowchart of the patient inclusion criteria.

From August 2015 to February 2020, 356 eligible breast cancer patients who underwent NSM or SSM followed by immediate reconstructive surgery were included in the study. Prophylactic mastectomy cases were not included. The exclusion criteria were as follows: (1) lack of postoperative follow-up in our institution (*n* = 8), (2) absence of postoperative MRI (*n* = 59), (3) absence of preoperative or pre-treatment MRI (*n* = 2), and (4) non-breast-origin tumors (*n* = 1, angiosarcoma). A total of 286 cases, including 9 cases of bilateral NSM or SSM with immediate reconstruction for the treatment of bilateral breast cancer, were finally enrolled (Figure 1).

### 2.2. Clinicopathologic Information 

The clinicopathologic information of the enrolled cases was reviewed using medical records and included age, follow-up period after surgery, and the mastectomy and reconstruction methods. In addition, the histologic type of tumor, tumor grade, stage (by AJCC 7th), hormone receptor status, human epidermal growth factor receptor 2 (HER2) status, Ki-67 index, and the presence of lymphovascular invasion (LVI) were reviewed based on surgical pathologic reports. The clinical stage was applied if the patient underwent neoadjuvant chemotherapy. Moreover, if patients underwent neoadjuvant chemotherapy, their hormone receptor status, HER2 status, and Ki-67 index were reviewed based on the biopsy pathology. 

Local recurrence was defined as a newly developed malignancy in the ipsilateral reconstructed breast. Newly diagnosed breast cancer of the contralateral breast, axillary lymph node metastasis, or distant metastasis were not assessed in this study. The recurrence interval was defined as the time between surgery and the diagnosis of recurrence. All recurrent lesions were pathologically confirmed through ultrasound-guided core-needle biopsy or surgical excision. 

### 2.3. MRI Protocols 

For postoperative MRI, routine dynamic contrast-enhanced (DCE) breast MRI was performed using the same protocol as that for pretreatment breast MRI. Images were obtained using 3-T MRI (Ingenia; Philips Medical Systems, Best, The Netherlands, Verio; Siemens Healthcare, Erlangen, Germany). The sequences of DCE MRI at our institution are as follows: (a) an axial turbo spin-echo T2-weighted imaging sequence with a repetition time msec/echo time msec of 3530/93, flip angle of 80°, 34 sections, a field of view of 350 mm, a matrix size of 576 × 403, one acquired signal, a section thickness of 4 mm, and an acquisition time of 2 min 28 s; and (b) pre-and post-contrast material administration axial T1-weighted fast low-angle shot three-dimensional volumetric interpolated brain examination sequences, 5.0/1.6, a flip angle of 12°, a section thickness of 1.2 mm, and an acquisition time of 1 min. The images were obtained before MRI and at 10, 70, 130, 190, 250, and 310 s after the injection of gadopentetate dimeglumine (0.1 mmol/kg, Gadovist; Bayer Schering Pharma, Berlin, Germany).

### 2.4. Imaging Surveillance after Surgery 

**Figure A1:**
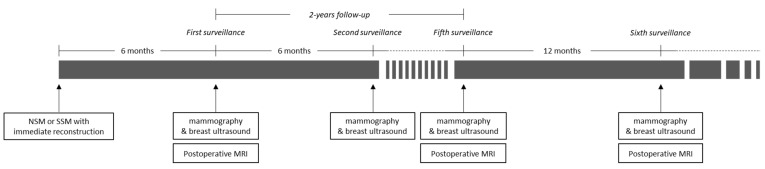
One of representative example of postoperative surveillance in our institution. As the diagram shows, first imaging surveillance will be performed at 6 months from surgery, and for two years, mammography and ultrasound will be performed with 6 months of interval. Postoperative MRI can be performed at 6 months or 12 months after surgery. If there is no suspicious result in postoperative MRI, it will be performed with 12 months of interval.

At our institution, most breast cancer patients underwent mammography of the contralateral (non-mastectomy) breast and ultrasound at 6 months after mastectomy as part of routine postoperative imaging surveillance. In addition, the first postoperative breast MRI scan was performed 6 months or 12 months after surgery in most cases. Although the first postoperative MRI can be delayed to after 12 months in some cases, it is performed at least once within 5 years after surgery. When postoperative MRI was performed as part of routine surveillance, contralateral mammography and ultrasound were also performed on the same day in most cases. Contralateral mammography and ultrasound were performed at intervals of 6 months or 12 months depending on the results of previous imaging surveillance. Postoperative MRI was also performed at intervals of 6 months or 12 months depending on the results of the previous MRI. At our institution, postoperative MRI was not included as part routine surveillance within 5 years after surgery if no disease recurrence was identified (Figure A1).

### 2.5. Imaging Analysis of Primary Cancer and Recurrent Lesions 

The imaging characteristics of primary breast cancer and recurrent lesions were reviewed by two breast expert radiologists with 8 and 25 years of experience in breast MRI. On pretreatment MRI, the location of the primary breast cancer, the type of lesion (mass, non-mass enhancement, or both), and the multifocality or multicentricity of the tumor were reviewed. Additionally, the background parenchymal enhancement and fibroglandular tissue were reviewed according to the American College of Radiology (ACR) Breast Imaging Reporting and Data System (BI-RADS) lexicon [30]. The imaging characteristics, including the size, location, and kinetic pattern, of recurrent lesions were evaluated using the postoperative MRI at the time of the diagnosis of recurrence. 

The lesion detectability rates using ultrasound and postoperative MRI for recurrent lesions were also investigated. We assessed the lesion detectability based on the reports which were initially written by radiologists with variable years of experience in breast imaging and confirmed by breast expert radiologists. The lesion detectability was considered positive when the lesion was not only identified in the imaging exam but also classified as BI-RADS category 4 or higher. In other words, if the lesion was classified as BI-RADS category 3 or less in the imaging exam, the lesion detectability was considered negative, even though the lesion had been found in the imaging exams. 

### 2.6. Statistical Analysis 

Patient characteristics were described as the mean, median, standard deviation (SD), range of continuous variables, and frequency of categorical variables. For the comparison of the characteristics between recurrence group and non-recurrence group, Fisher’s exact test or the chi-square test, for the categorical variables, and Student’s *t*-test or the Mann–Whitney U test, for the continuous variables, were used. Before the *t*-test, the Kolmogorov–Smirnov test was performed for the normality. The chi-square test was used for the comparison of the proportions, including the detection rate of each imaging modality for recurrent lesions. All statistical analyses were conducted using SPSS version 23 (IBM Corp., Armonk, NY, USA).

## 3. Results

### 3.1. Characteristics of the Patients

Among the 286 cases, invasive ductal carcinoma of no specific type (NST) (*n* = 197) was the most common histologic type, followed by DCIS (*n* = 45) and invasive lobular carcinoma (*n* = 21). Stage II (*n* = 108) was the most common stage, and luminal type (*n* = 230) was the most common subtype. Every enrolled patient underwent standard treatment for breast cancer according to their stage and tumor subtype. One hundred and ninety-two patients underwent NSM (67.1%), and ninety-four patients underwent SSM (32.9%), which showed a significant difference (*p* < 0.001). However, there was no significant difference between the recurrence group and non-recurrence group in terms of the operative method of mastectomy. The nipple resection margin was negative in all the NSM cases. One hundred and fifty-five patients underwent flap reconstruction (54.2%), and one hundred and thirty-three patients underwent implant bag reconstruction (45.8%), which showed no significant difference (*p* = 0.05). There was no significant difference between the recurrence and non-recurrence groups in terms of the method of reconstruction. The details of the characteristics of patients are described in Table 1. 

### 3.2. Imaging Characteristics of Primary Breast Cancer

There was no significant difference between the recurrence group and non-recurrence group in terms of the laterality of the primary tumor (*p* = 0.945), lesion type (*p* = 0.788), and the grade of background parenchymal enhancement (*p* = 0.493). However, the recurrence group showed a higher rate of multifocality or multicentricity of their tumors (20 cases of 22 cases, 90.9%, *p* < 0.001) compared to the non-recurrence group. There was no fatty nor scattered fibroglandular tissue pattern identified in the recurrence group, which showed a significant difference between the recurrence and non-recurrence groups (*p* = 0.037) (Table 2). 

### 3.3. Characteristics of the Recurrent Lesions 

**Figure 2:**
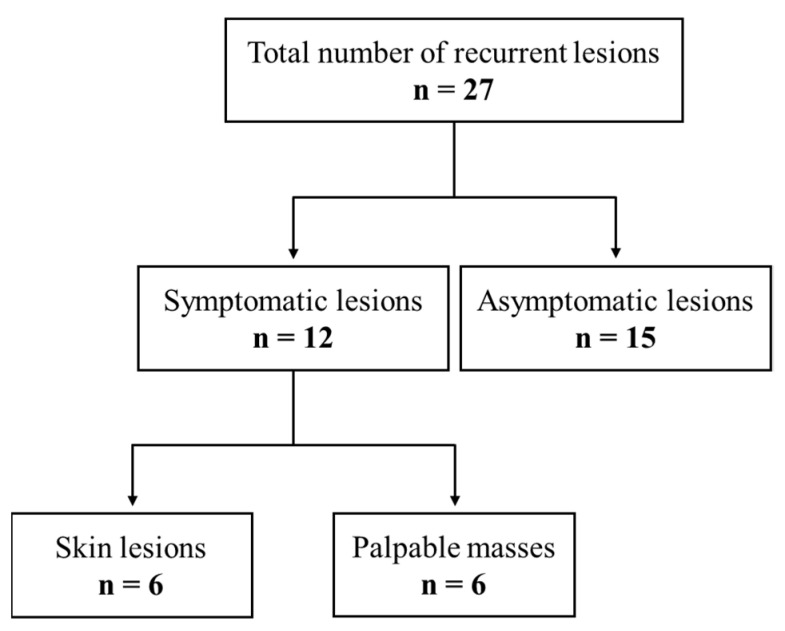
Diagram of the recurrent lesions.

Of the 286 cases, 22 cases showed recurrence in the reconstructed breast, with a median recurrence interval of 12 months (range: 3 to 51). In 4 cases, as multiple recurrent lesions were pathologically confirmed, there were 27 recurrent lesions in 22 recurrent cases (Figure 2). Regardless of the lesion detectability rates of the imaging modalities, all recurrent mass lesions were identified for biopsy or localization for excision through target ultrasound performed by the breast expert radiologists.

The histology of the recurrent lesions was same as the histology of the primary breast cancers, except for one lesion. This one lesion was diagnosed as invasive ductal carcinoma, in contrast to the histology of the primary breast cancer, which was mucinous carcinoma. 

Of 27 recurrent lesions, 12 lesions were accompanied by symptoms, and 15 lesions were not accompanied by symptoms. There were six cases of skin recurrence, and they were all detected through physical exams and not imaging exams. 

The other twenty-one lesions were manifested as masses in reconstructed breasts. All recurrent mass lesions were superficially located in the reconstructed breasts, and there was no lesion located in the deep tissue adjacent to the chest wall. The recurrent mass lesions showed fast enhancement on early dynamic phase, except for one lesion, and most of the lesions showed washout (13 of 21, 61.9%) or plateau (7 of 21, 33.3%) patterns on delayed phase. The one lesion which did not show early fast enhancement showed a persistent enhancement pattern on delayed phase. 

The median recurrence interval of the symptomatic mass lesions was 8 months (range: 3 to 15 months), and the mean recurrence interval was 10.4 months (SD ± 5.8). The median recurrence interval of the asymptomatic lesions was 12 months (range, 5 to 51 months), and the mean recurrence interval was 21.7 months (SD ± 17.7). There was no significant difference in the median recurrence interval between lesion types (*p* = 0.198). However, the mean recurrence interval of the asymptomatic lesions was significantly longer than that of symptomatic lesions (*p* < 0.001) 

The mean size of the recurrent mass lesions was 0.8 cm (SD ± 0.5). The mean size of the symptomatic lesions was 1.2 cm (SD ± 0.7), and that of the asymptomatic lesions was 0.7 cm (SD ± 2.7). The mean size of the symptomatic lesions was significantly larger than that of the asymptomatic lesions (*p* = 0.004) (Table 3). 

The lesion detectability rates by the imaging modalities for recurrent mass lesions was assessed using initial reports of the imaging exams (Table 4). Since postoperative MRI was performed after ultrasound in every recurrent case, radiologists who performed the ultrasounds were not aware of the MRI findings when they performed the ultrasound. Of the 21 recurrent mass lesions, MRI detected 20 lesions (95.2%), excepting one lesion. The one recurrent lesion was identified by MRI, but it was classified as BI-RADS category 3; thus, the MR detectability of this lesion was considered negative. 

Using ultrasound, eight lesions (38.1%) were detected, and all these lesions were also detected in MRI. Of the 12 lesions not detected by ultrasound, 8 lesions were not identified by ultrasound at all, and 4 lesions were identified but classified as BI-RADS category 2 or higher. Regardless of the presence of symptoms, the mean size of the recurrent mass lesions detected by both ultrasound and MRI (*n* = 8, 1.1 ± SD 0.6 cm) was significantly larger than that of lesions detected only by MRI (*n* = 12, 0.7 ± SD 0.3 cm, *p* = 0.025).

**Figure 3:**
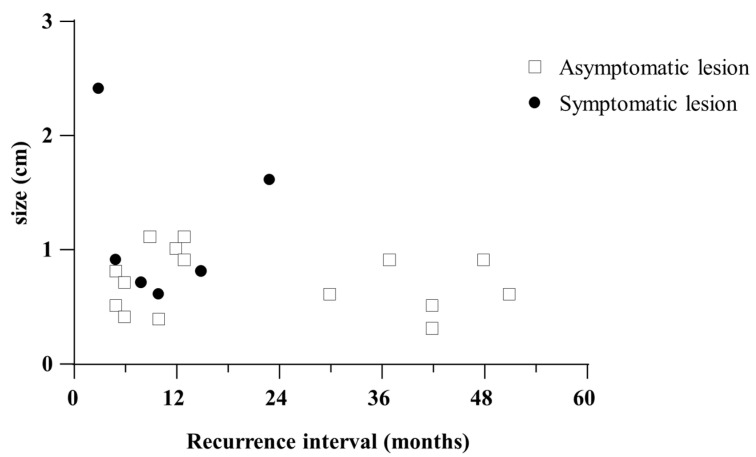
Distribution of the recurrent mass lesions according to size and recurrence interval.

Figure 3 shows the distribution of the recurrent mass lesions according to size and recurrence interval. Asymptomatic lesions show a wide range of recurrence intervals, but unlike symptomatic lesions, which all occurred within 24 months, there were recurrent lesions that occurred after 24 months. 

The distribution of the recurrence intervals and sizes of recurrent mass lesions is shown above. From this graph, we can see that most of the recurrent lesions were diagnosed within 12 months after surgery, regardless of the presence of symptoms and size. The asymptomatic lesions showed a tendency to have a recurrence interval longer than 24 months from the time of surgery, with a smaller size (<1 cm), whereas symptomatic lesions showed a tendency to have a shorter recurrence interval, with a larger size (>1 cm).

#### 3.3.1. Lesion Detectability Rates of the Imaging Modalities for Symptomatic Recurrence 

Of the 27 recurrent lesions, 12 lesions were accompanied by symptoms. Six manifested as skin lesions, and the other six were palpable lesions. Six cases of skin lesions were confirmed as skin metastasis by punch-biopsy. None were all detected upon imaging surveillance, including ultrasound and postoperative MRI (Table A1). 

**Figure 4:**
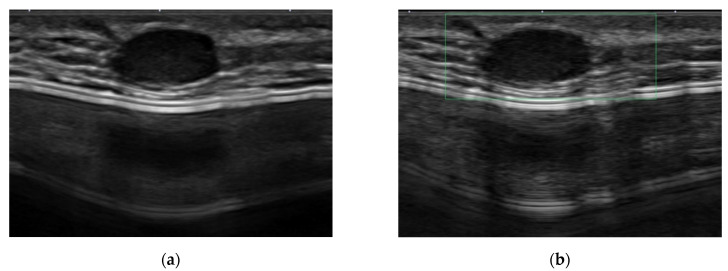

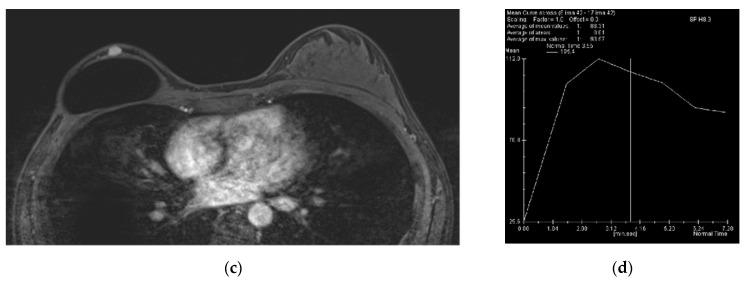
Symptomatic recurrent case mimicking a benign epidermal inclusion cyst. A 27-year-old female patient who underwent right-breast skin-sparing mastectomy with reconstruction with a silicone implant bag for mucinous carcinoma (grade 2, luminal type) 48 months ago. (**a**,**b**) Using ultrasound as a method of routine surveillance, an oval circumscribed hypoechoic mass with posterior enhancement was noted in the reconstructed breast, where the patient complained that a palpable lump was present. Since the mass is accompanied by skin tract and showed no vascularity on the Doppler scan, it was considered to be an epidermal inclusion cyst. (**c**,**d**) At postoperative MRI, the mass showed fast enhancement on the early dynamic phase fat-saturated T1-weighted image with a washout kinetic pattern. Based on the finding of the MRI scan, the category of the mass was upgraded to category 4, and it was confirmed as invasive ductal carcinoma by surgical excision.

The other six symptomatic lesions manifested as palpable lumps in the reconstructed breast. Postoperative MRI detected all symptomatic mass lesions as suspicious lesions. Using ultrasound, four lesions (66.7%) were detected as suspicious lesions, while the other two were classified as BI-RADS category 2 and 3 lesions, respectively. The lesion classified as BI-RADS category 2 was initially considered as an epidermal inclusion cyst by ultrasound (Figure 4), and the lesion category 3 was considered as fat necrosis. However, both lesions showed a suspicious enhancement in MRI and were upgraded to BI-RADS category 4. The lesion detectability rates of ultrasound and postoperative MRI for symptomatic recurrent mass lesions showed no significant difference (*p* = 0.140.) 

#### 3.3.2. Lesion Detectability Rates of the Imaging Modalities for Asymptomatic Recurrence

Fifteen lesions were not accompanied by symptoms. All fifteen lesions were mass lesions, which were detected by imaging surveillance, except for one lesion. The one lesion, previously mentioned as the lesion not detected in MRI, was also not identified by routine ultrasound. It was detected by targeted ultrasound performed by the breast expert radiologist and confirmed as recurrence by ultrasound-guided core-needle biopsy. The characteristics of the asymptomatic recurrent lesions are described Table A1. 

**Figure 5:**
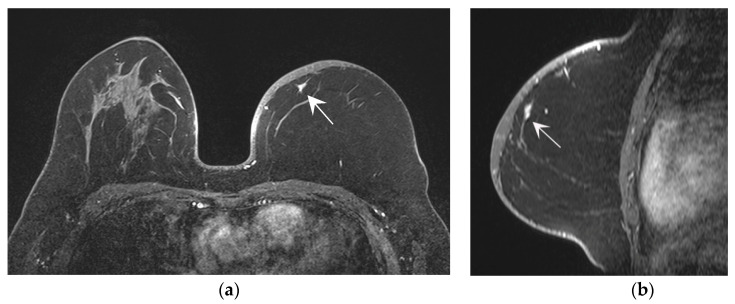

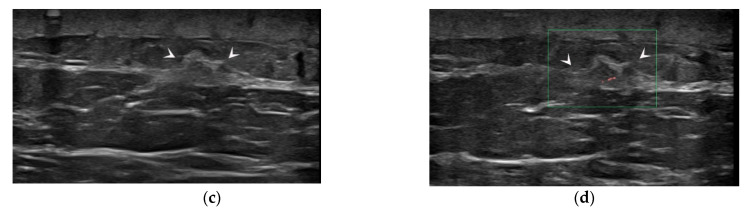
Asymptomatic recurrence after nipple-sparing mastectomy with flap reconstruction. A 42-year-old female patient who underwent nipple-sparing mastectomy with deep inferior epigastric artery perforator flap reconstruction for invasive ductal carcinoma (grade 2, luminal type) 48 months ago. (**a**,**b**) Using postoperative MRI as a method of routine postoperative surveillance, an irregularly shaped enhancing focus was noted in the inner region of the left reconstructed breast (arrow). It was a newly noted lesion, according to a comparison with the previous routine surveillance MRI from 12 months earlier. (**c**,**d**) Using targeted ultrasound to detect the enhancing lesion, an irregularly shape hyperechoic lesion with indistinct margins confused with the surrounding tissue was noted at the corresponding site (arrow heads). It showed intra-lesional vascularity on the Doppler scan, and it was confirmed as recurrent breast cancer by ultrasound-guided core-needle biopsy.

Ultrasound detected four lesions (26.7%) as suspicious lesions, and they were all also detected by MRI. MRI detected fourteen lesions (93.3%) as suspicious lesions, and it showed a significantly higher detection rate (*p* < 0.001) than ultrasound. Ten lesions were detected as suspicious lesions only by MRI. Of these 10 lesions, 3 were identified by ultrasound but considered to be fat necrosis and classified as BI-RADS category 3. For the other seven lesions not identified by routine ultrasound, targeted ultrasound for biopsy or localization for excision was performed by breast expert radiologists, and they were all detected by targeted ultrasound and pathologically confirmed as recurrent lesions (Figure 5).

## 4. Discussion

In our study, ultrasound and postoperative MRI were mainly applied for the purpose of surveillance after NSM or SSM with reconstruction surgery. In addition to skin recurrence, MRI showed a high detectability rate for all recurrent lesions, overall, but showed a higher detectability than ultrasound for asymptomatic lesions, particularly small-sized (<1 cm) lesions. Considering the high sensitivity of MRI in detecting recurrence after breast cancer surgery [31,32], this is not a particularly novel result. However, in our study, MRI not only identified recurrent lesions efficiently, but also helped to diagnose lesions that were underestimated by ultrasound as recurrence. 

Physical examination, which is recommended as a surveillance method after conventional mastectomy, has also been suggested as a surveillance method for reconstructed breasts in some previous studies [19,20]. The authors advocated the use of physical examination because most cases of local recurrence in reconstructed breasts occurred in a subcutaneous location or the skin, making it amenable for clinical detection. All recurrent lesions except skin lesions in our study also had a superficial location in the reconstructed breast, as in previous studies. In the case of skin recurrence, physical examination is important because, here, it was found only through physical examination rather than imaging exams. However, the number of symptomatic lesions manifested as palpable lumps was 40% (6 of 15), which was not half. In other words, there may be limitations to detecting recurrence only by the physical examination of reconstructed breasts. 

There are several previous studies on postoperative surveillance after mastectomy with autologous reconstruction. Most of the studies suggested annual mammography as the surveillance modality [18,33]. However, other studies have reported the insufficient evidence for the effectiveness of annual mammography in autologous reconstructed breast [20,34]. Annual mammography can be viewed as a reasonable choice of modality due to its cost-effectiveness and accessibility. However, mammography of autologous reconstructed breast has limitations, including the image quality due to architectural distortion. 

Ultrasound is often performed as the imaging modality of choice for the evaluation of palpable masses in patients with reconstructed breasts. Ultrasound is applied in various situations after breast surgery, as well as for evaluation of symptomatic lesions. Some previous studies demonstrated the effectiveness of ultrasound after mastectomy, with a high degree of sensitivity in the detection of early recurrence [35,36]. However, in our study, limitations of ultrasound were revealed in several cases. Of six symptomatic mass lesions, four lesions were detected by ultrasound. The other two lesions were considered to be benign lesions, although they were identified by ultrasound. These cases showed the limitation of ultrasound, in the sense that recurrent lesions cannot be distinguished by ultrasound when recurrent lesions are mimicking benign lesions. There were also eight recurrent lesions that were only detected by postoperative MRI and not identified by ultrasound at all. However, they were detected by targeted ultrasound performed by a breast expert. These cases showed the typical limitation of ultrasound, being operator dependency. 

In our study, postoperative MRI showed a rate of detectability that was not high not only for all recurrent lesions, but also for asymptomatic and small-sized recurrent lesions. In addition, the mean size of the asymptomatic lesions was also smaller (<1 cm) than that of symptomatic lesions. Based on this result, MRI can be useful for detecting small (<1 cm) asymptomatic recurrent lesions, without a delay in diagnosis. Since it is proven that the early detection of recurrent breast cancer in the asymptomatic phase increases the patient’s survival rate [37], the early detection of asymptomatic recurrent lesions using postoperative MRI can also be helpful for ensuring a patient’s survival.

MRI can also be useful in symptomatic recurrent cases. There were two cases diagnosed as recurrence by MRI, even though the lesions were accompanied by symptoms and identified by ultrasound. In addition, another three recurrent lesions were additionally detected by MRI in two symptomatic recurrent cases, which were already confirmed. These were additionally excised through ultrasound-guided localization by breast expert radiologists and finally confirmed as recurrence, leading to oncologically complete surgery. 

Several previous studies have proven the usefulness of postoperative MRI after breast cancer surgery [31,32,38,39]. However, due to insufficient evidence and its high cost, MRI’s use as a routine postoperative surveillance method is controversial [23,40,41]. Moreover, clinicians or radiologists may be confused about the indication of surveillance MRI or the surveillance interval of MRI, because there is no guideline on the use of MRI for surveillance after mastectomy with reconstruction surgery. One previous study recommended postoperative MRI only for high-risk patients [42], and Park et al. suggested postoperative MRI as a surveillance tool for patients who have a personal history of breast cancer, and they argued that postoperative MRI can be more effective following 3 years after surgery [43]. 

In our study, though asymptomatic recurrent mass lesions were diagnosed with a wide range of recurrence intervals, more than half of the asymptomatic recurrent lesions (9 of 15) were detected by postoperative MRI within 24 months (Figure 2), and all asymptomatic cases were diagnosed within 5 years. Moreover, local recurrence was more frequently diagnosed when the patients had dense breast parenchyma and the primary cancer showed multifocality or multicentricity on preoperative MRI. From these results, we can suggest that the first postoperative MRI should performed within 24 months after surgery, especially when the patients have dense breast parenchyma or multifocal/multicentric primary cancers. If postoperative MRI cannot be performed within 24 months after surgery, we suggest that postoperative MRI be performed within at least 5 years after surgery. However, the indication and surveillance interval of postoperative MRI should be further investigated in future studies. 

There are several limitations of our study. Firstly, this study is a retrospective single-center study, which may weaken the representativeness of the data. Secondly, the different time intervals between the first postoperative MRI and surgery for each patient can be considered as a limitation. Finally, a comparison between the effectiveness of mammography and MRI in recurrence detection was not achieved, because mammography of the reconstructed breast is not routinely performed in our institution. In addition, there was no comparison with patients who did not undergo postoperative MRI after mastectomy and reconstruction. Although there are several limitations, we have included a relatively large data set, and our study can be viewed as meaningful in the sense that we have investigated the usefulness of MRI. 

In conclusion, postoperative MRI can be useful for detecting local recurrence in reconstructed breast after NSM or SSM, especially when the recurrent lesions are small (<1 cm) and asymptomatic. In future studies, it will be necessary to identify long-term survival rates and examine how the early detection of asymptomatic recurrent lesions affected patient survival after NSM or SSM with reconstructive surgery, and a comparison with patients who did not recieve postoperative MRI will be required. 

## Figures and Tables

**Table 1:** Patient characteristics.

	Total (*n* = 286)	Non-Recurrence (*n* = 264)	Recurrence (*n* = 22)	*p* Value
**Clinical information**				
**Age (mean ± SD ^1^)**	46.0 (± 8.3)	46.2 (± 8.1)	43.0 (± 9.7)	0.352
**Operation**				0.716
nipple-sparing mastectomy (NSM)	192 (67.1%)	178 (67.4%)	14 (63.6%)	
skin-sparing mastectomy (SSM)	94 (32.9%)	86 (32.6%)	8 (36.4%)	
**Reconstruction**				0.081
flap reconstruction	155 (54.2%)	147 (55.7%)	8 (36.4%)	
implant bag reconstruction	131 (45.8%)	117 (44.3%)	14 (63.6%)	
**Follow-up period (months)**				0.766
median (range)	30 (8–63)	30 (8–63)	30 (9–51)	
**Treatment**				
Neoadjuvant chemotherapy				0.37
yes	40 (14.0%)	36 (13.6%)	4 (18.2%)	
no	246 (86%)	228 (86.4%)	18 (81.8%)	
Adjuvant radiation therapy				0.523
yes	71 (24.8%)	66 (25.0%)	5 (22.7%)	
no	215 (75.2%)	198 (75.0%)	17 (77.3%)	
Adjuvant chemotherapy				0.321
yes	133 (46.5%)	125 (47.3%)	8 (36.4%)	
no	153 (53.5%)	139 (52.7%)	14 (63.6%)	
Adjuvant endocrine therapy				0.834
yes	226 (79.0%)	209 (79.2%)	17 (77.3%)	
no	60 (21.0%)	55 (20.8%)	5 (22.7%)	
Adjuvant target therapy				0.176
yes	54 (18.9%)	52 (19.7%)	2 (9.1%)	
no	232 (81.1%)	212 (80.3%)	20 (90.9%)	
**Pathologic information**				
**TNM Staging**				
T				0.763
0	35 (12.2%)	34 (12.9%)	1 (4.5%)	
1	104 (36.4%)	94 (35.6%)	10 (45.5%)	
2	111 (38.8%)	103 (39.0%)	8 (36.4%)	
3	35 (12.2%)	32 (12.1%)	3 (13.6%)	
4	1 (0.4%)	1 (0.4%)	0 (0.0%)	
*n*				0.831
0	169 (59.1%)	154 (58.3%)	15 (68.2%)	
1	76 (26.6%)	71 (26.9%)	5 (22.7%)	
2	20 (7.0%)	19 (7.2%)	1 (4.5%)	
3	21 (7.3%)	20 (7.6%)	1 (4.5%)	
**Stage**				0.298
stage 0	35 (12.2%)	34 (12.9%)	1 (4.5%)	
stage I	83 (29.0%)	73 (27.6%)	10 (45.5%)	
stage II	108 (37.8%)	101 (38.3%)	7 (31.8%)	
stage III	60 (21.0%)	56 (21.2%)	4 (18.2%)	
**Cancer histology**				0.553
ductal carcinoma in situ	45 (15.7%)	42 (15.9%)	3 (13.6%)	
invasive ductal carcinoma, NST ^2^	197 (69.0%)	180 (68.2%)	17 (77.3%)	
invasive lobular carcinoma	21 (7.3%)	21 (8.0%)	0 (0.0%)	
others	23 (8.0%)	21 (8.0%)	2 (9.1%)	
**Lymphovascular invasion**				0.562
positive	76 (26.6%)	69 (24.1%)	7 (31.8%)	
negative	210 (73.4%)	195 (73.9%)	15 (68.2%)	
**Tumor size (cm)**				
**Estrogen receptor**				0.766
positive	228 (79.7%)	211 (79.9%)	17 (77.3%)	
negative	58 (20.3%)	53 (20.1%)	5 (22.7%)	
**Progesterone receptor**				0.481
positive	213 (74.5%)	198 (75.0%)	15 (68.2%)	
negative	73 (25.5%)	66 (25.0%)	7 (31.8%)	
**HER2 ^3^**				0.912
positive	88 (30.8%)	81 (30.7%)	7 (31.8%)	
negative	198 (69.2%)	183 (69.3%)	15 (68.2%)	
**Ki 67 index**	32.7 (± 20.4)	32.4 (± 20.5)	36.6 (± 18.9)	0.963
**Subtype**				0.829
luminal	230 (80.4%)	213 (80.7%)	17 (77.3%)	
HER2	40 (14.0%)	36 (13.6%)	4 (18.2%)	
triple negative	16 (5.6%)	15 (5.7%)	1 (4.5%)	

^1^ Standard deviation; ^2^ no special type; ^3^ human epidermal growth factor receptor 2.

**Table 2:** MRI characteristics of primary breast cancer.

	Total (*n* = 286)	Non-Recurrence (*n* = 264)	Recurrence (*n* = 22)	*p* Value
**Tumor location**				0.945
right	128 (44.8%)	118 (44.7%)	10 (45.5%)	
left	158 (55.2%)	146 (55.3%)	12 (54.5%)	
**Lesion type**				0.788
mass	73 (25.5%)	66 (25.0%)	7 (31.8%)	
non-mass enhancement	103 (36.0%)	96 (36.4%)	7 (31.8%)	
both	110 (38.5%)	102 (38.6%)	8 (36.4%)	
**Multifocality/multicentricity**				<0.001
yes	206 (72.0%)	186 (70.5%)	20 (90.9%)	
no	80 (28.0%)	78 (29.5%)	2 (9.1%)	
**Background parenchymal enhancement**				0.493
minimal	119 (41.6%)	107 (40.5%)	12 (54.5%)	
mild	71 (24.8%)	68 (25.8%)	3 (13.6%)	
moderate	63 (22.0%)	59 (22.3%)	4 (18.2%)	
marked	33 (11.6%)	30 (11.4%)	3 (13.6%)	
**Fibroglandular tissue**				0.037
fatty	0 (0.0%)	0 (0.0%)	0 (0.0%)	
scattered	12 (4.2%)	12 (4.6%)	0 (0.0%)	
heterogenous	194 (67.8%)	183 (69.3%)	11 (50.0%)	
extreme	80 (28.0%)	69 (26.1%)	11 (50.0%)	

**Table 3:** Characteristics of the recurrent lesions according to the presence of symptoms.

	Total (*n* = 27)	Symptomatic (*n* = 12)	Asymptomatic (*n* = 15)	*p* Value
Lesion type				0.002
mass	21 (77.8%)	6 (50.0%)	15 (100.0%)	
skin	6 (22.2%)	6 (50.0%)	0 (0.0%)	
**Recurrence interval (months)**				
median (range)	12 (3–51)	8 (3–15)	12 (5–51)	0.198
mean (SD ^1^)	16.7 (± 14.67)	10.4 (± 5.82)	21.7 (± 17.69)	<0.001
**Detection modality**				0.001
physical examination	6 (22.2%)	6 (50.0%)	0 (0.0%)	
ultrasound	9 (33.3%)	5 (41.7%)	4 (26.7%)	
MRI	12 (44.4%)	1 (8.3%)	11 (73.3%)	
**Tumor size (MRI) (cm)**				
mean (SD)	0.8 (± 0.46)	1.2 (± 0.70)	0.7 (± 2.67)	0.004
**Primary cancer histology**				0.323
ductal carcinoma in situ	3 (11.1%)	1 (8.3%)	3 (20.0%)	
invasive ductal carcinoma, NST ^2^	20 (74.1%)	10 (83.3%)	9 (60.0%)	
invasive lobular carcinoma	0 (0.0%)	0 (0.0%)	0 (0.0%)	
others	4 (14.8%)	1 (8.3%)	3 (20.0%)	
**Primary cancer subtype**				0.905
luminal	17 (63.0%)	8 (66.7%)	9 (60.0%)	
HER2	9 (33.3%)	3 (25.0%)	6 (40.0%)	
triple negative	1 (3.7%)	1 (8.3%)	0 (0.0%)	
**Stage**				0.152
stage 0	3 (11.1%)	1 (8.3%)	2 (13.3%)	
stage I	13 (48.2%)	4 (33.3%)	9 (60.0%)	
stage II	10 (37.0%)	6 (50.0%)	4 (26.7%)	
stage III	1 (3.7%)	1 (8.3%)	0 (0.0%)	
**Adjuvant therapy**				
radiation therapy				0.067
yes	5 (18.5%)	5 (41.7%)	0 (0.0%)	
no	22 (81.5%)	7 (58.3%)	15 (100.0%)	
chemotherapy				0.792
yes	10 (37.0%)	4 (33.3%)	6 (40.0%)	
no	17 (63.0%)	8 (66.7%)	9 (60.0%)	
endocrine therapy				0.614
yes	22 (81.5%)	9 (75.0%)	13 (86.7%)	
no	5 (18.5%)	3 (25.0%)	2 (13.3%)	
target therapy				0.829
yes	3 (11.1%)	1 (8.3%)	2 (13.3%)	
no	24 (88.9%)	11 (91.7%)	13 (86.7%)	

^1^ Standard deviation; ^2^ no special type.

**Table 4:** Detectability of the recurrent mass lesions by imaging modality.

Imaging Modality	Total (*n* = 21)	Symptomatic (*n* = 6)	Asymptomatic (*n* = 15)
MRI	20 (95.2%)	6 (100.0%)	14 (93.3%)
Ultrasound	8 (38.1%)	4 (66.7%)	4 (26.7%)
*p* value	<0.001	0.140	<0.001

## Data Availability

All data generated and analyzed during this study are included in this published article. Raw data supporting the findings of this study are available from the corresponding author on request.

## Appendix A

**Table A1:** Characteristics of asymptomatic recurrent lesions.

Clinical Characteristics			Surgery		Surveillance		Characteristics of Primary Cancer						Adjuvant Therapy			Imaging Characteristics		
Case Number	Lesion Number	Age	Mastectomy	Reconstruction	Detection Modality	Follow-Up Period (Months)	Histology	Grade	ER ^8^	PR ^9^	HER2 ^10^	Stage	Chemotherapy	Endocrine	Target Agent	Early Kinetics	Delay Kinetics	Size (cm)
No. 1	No. 1 ^†^	29	NSM ^1^	implant	Targeted US ^3^	51	IDC, NST	2	+ ^11^	+	− ^12^	IA	no	yes	no	fast	washout	0.6
No. 2	No. 2	53	NSM	flap	MRI	29	Metaplastic	3	+	−	−	IA	yes	yes	no	fast	plateau	1.1
No. 3	No. 3	47	NSM	flap	MRI	48	IDC ^5^, NST ^6^	2	+	+	+	IIIA	yes	yes	yes	fast	washout	0.5
	No. 4		NSM	flap	MRI	48	IDC, NST	2	+	+	+		yes	yes	yes	medium	persistent	0.3
No. 4	No. 5	47	NSM	flap	US	35	IDC, NST	2	−	−	+	IA	no	no	no	fast	washout	1.1
No. 5	No. 6	42	NSM	implant	MRI ^4^	39	IDC, NST	3	+	+	−	IA	no	yes	no	fast	washout	0.9
No. 6	No. 7	46	SSM ^2^	flap	MRI	49	IDC, NST	2	+	+	−	IIA	no	yes	no	fast	plateau	0.6
No. 7	No. 8	38	NSM	flap	MRI	48	IDC, NST	2	+	+	−	IIB	yes	yes	no	fast	plateau	0.9
No. 8	No. 9	26	SSM	implant	US	36	Mucinous	2	+	+	−	IA	no	yes	no	fast	washout	0.8
	No. 10		SSM	implant	MRI	36	Mucinous	2	+	+	−	IA	no	yes	no	fast	washout	0.5
No. 9	No. 11	36	NSM	implant	US	25	DCIS ^7^	2	+	+	+	0	yes	yes	no	fast	washout	0.7
	No. 12		NSM	implant	MRI	25	DCIS	2	+	+	+	0	yes	yes	no	fast	plateau	0.4
	No. 13		NSM	implant	US	25	DCIS	2	+	+	+	0	yes	yes	no	fast	washout	0.4
No. 10	No. 14	40	SSM	implant	US	29	IDC, NST	3	−	−	+	0	no	no	no	fast	plateau	1
No. 11	No. 15	44	SSM	flap	MRI	25	IDC, NST	2		+	−	IIA	no	yes	no	fast	plateau	0.9

^1^ Nipple sparing mastectomy; ^2^ Skin sparing mastectomy; ^3^ Ultrasound; ^4^ Magnetic resonance imaging; ^5^ Invasive ductal carcinoma; ^6^ No special type; ^7^ Ductal carcinoma in situ; ^8^ Estrogen receptor; ^9^ Progesterone receptor; ^10^ Human epidermal growth factor receptor 2; ^11^ “+” sign means positive for receptors; ^12^ “−” sign means negative for receptors; ^†^ This lesion was not detected by both MRI (category 3) nor routine ultrasound (not identified), but detected by targeted ultrasound performed by breast expert radiologist.
